# Characterization of microRNAs during Embryonic Skeletal Muscle Development in the Shan Ma Duck

**DOI:** 10.3390/ani10081417

**Published:** 2020-08-14

**Authors:** Chuan Li, Ting Xiong, Mingfang Zhou, Lei Wan, Suwang Xi, Qiuhong Liu, Yi Chen, Huirong Mao, Sanfeng Liu, Biao Chen

**Affiliations:** College of Animal Science and Technology, Jiangxi Agricultural University, Nanchang 330045, China; lichuan0122@126.com (C.L.); xt970123@163.com (T.X.); bighawkin@sina.com (M.Z.); gaosan0211@163.com (L.W.); xswjxau@163.com (S.X.); liuqiuhong157@163.com (Q.L.); elie1448613389@163.com (Y.C.); huirongmjxau@126.com (H.M.); lsf3318@jxau.edu.cn (S.L.)

**Keywords:** microRNA, skeletal muscle, muscle development, Shan Ma duck, high throughput sequencing

## Abstract

**Simple Summary:**

It is of great commercial interest to elucidate the genetic mechanisms associated with skeletal muscle development in the duck. In this study, we performed high throughput microRNA (miRNA) sequencing to identify the candidate miRNAs during two developmental stages of duck embryonic breast muscle. We detected 1091 miRNAs and 109 of them were differentially expressed between embryonic day 13 (E13) and E19. We also predicted the target genes of the differentially expressed miRNAs and subsequently analyzed the enriched gene ontology (GO) terms and Kyoto Encyclopedia of Genes and Genomes (KEGG) signaling pathways, and finally constructed a protein–protein interaction (PPI) network with the target genes. Luciferase reporter assay showed that the growth-related genes, *Fibroblast growth factor receptor like 1* (*FGFRL1*) and *Insulin like growth factor 2 mRNA binding protein 1* (*IGF2BP1*), were target genes of miR-214-5p. These results can supplement the duck miRNA database and provide several candidate miRNAs for future studies on the regulation of embryonic skeletal muscle development.

**Abstract:**

Poultry skeletal muscle provides high quality protein for humans. Study of the genetic mechanisms during duck skeletal muscle development contribute to future duck breeding and meat production. In the current study, three breast muscle samples from Shan Ma ducks at embryonic day 13 (E13) and E19 were collected, respectively. We detected microRNA (miRNA) expression using high throughput sequencing following bioinformatic analysis. qRT-PCR validated the reliability of sequencing results. We also identified target prediction results using the luciferase reporter assay. A total of 812 known miRNAs and 279 novel miRNAs were detected in six samples; as a result, 61 up-regulated and 48 down-regulated differentially expressed miRNAs were identified between E13 and E19 (|log2 fold change| ≥ 1 and *p* ≤ 0.05). Enrichment analysis showed that target genes of the differentially expressed miRNAs were enriched on many muscle development-related gene ontology (GO) terms and Kyoto Encyclopedia of Genes and Genomes (KEGG) pathways, especially mitogen-activated protein kinase (MAPK) signaling pathways. An interaction network was constructed using the target genes of the differentially expressed miRNAs. These results complement the current duck miRNA database and offer several miRNA candidates for future studies of skeletal muscle development in the duck.

## 1. Introduction

Skeletal muscle forms a large proportion of the body of animals. Food conversion ratio and meat production are important economic indicators in livestock and the growth and development of skeletal muscle has an appreciable impact on meat production. Therefore, an exploration of the molecular mechanisms related to skeletal muscle growth and development is important to human food security. The consumption of poultry meat has shown a notable increase since the emergence of African swine fever and the increase in pork prices in China (http://data.stats.gov.cn/). Skeletal muscle development can be divided into two stages: myoblast proliferation and the hypertrophy of myotubes. In the embryonic stage, myoblasts proliferate and differentiate into multi-nucleated myotubes; subsequently, myotubes further differentiate into mature muscle fibers [1]. The number of muscle fibers becomes fixed after their formation during the embryonic stage. After hatching, satellite cells fuse with muscle fibers, resulting in muscle fiber enlargement or hypertrophy [2]. There are many molecules that regulate the growth and development of poultry muscle, including protein-coding genes and non-coding RNAs [3,4,5]. Embryonic day 19 (E19) is a crucial stage of duck breast muscle development. Myogenic factor 6 (MRF4) and myogenin (MyoG) have the highest expression level and myostatin (MSTN) has the lowest expression level in this stage, the breast muscle weight is nearly constant from E19 to hatching [6].

MicroRNAs (miRNAs) are single-stranded non-coding RNA with a length of 18–26 nucleotides (nt). These can promote the degradation of messenger RNA (mRNA) and inhibit its translation by complementary pairing with the 3’ untranslated region (3’UTR) of the target mRNA, thus exerting post-transcriptional regulation of gene expression [7]. miRNAs not only regulate gene translation in cells, but also play a messenger role intercellularly or in the circulatory system [8,9]. Furthermore, miRNAs can be communicators between different types of RNA that share the same miRNA response elements [10]. In addition, the expression of miRNAs leads to special temporal and spatial characteristics [11]. During skeletal muscle development, some miRNAs are specifically expressed in skeletal muscle, while others show significant differential expression before and after skeletal muscle differentiation [12]. miRNAs affect skeletal muscle development mainly through the regulation of differentiation factors and growth-related pathways [13,14]. There are some myo-miRNAs, such as miR-1, miR-133a, miR-133b, and miR-206 that are abundantly expressed and play important roles during poultry myogenesis [15,16,17].

miRNAs in embryonic breast muscle of Pekin duck were profiled in E13, E19 and E27 in 2014 [18]. In this current study, we analyzed the expression profile of miRNAs with a new sequencing platform from Shan Ma duck breast muscles at E13 and E19. The Shan Ma duck is a small bodied, indigenous duck breed in China. E13 and E19 samples represent myoblasts in skeletal muscle which did not differentiate and differentiated, respectively. Using bioinformatics analysis, we identified differentially expressed miRNAs between the two stages and analyzed the potential functions of these miRNAs. We suggest that this information is a valuable addition to the duck miRNA database and provides greater insight into the molecular mechanisms of duck muscle development.

## 2. Materials and Methods

### 2.1. Ethics Statement

All experimental procedures in this study followed the ethical standards and regulations of Jiangxi Agricultural University (JXAULL-2017002). All efforts were made to minimize any pain experienced by the experimental ducks.

### 2.2. Sample Collection, Library Construction, and miRNA Sequencing

A total of 30 fertile Shan Ma duck eggs from the same batch were purchased from Jiangxi Tianyun duck breeding farm (Nanchang, Jiangxi, China): 15 embryos were at embryonic day 13 (E13) and 15 embryos at E19. Breast muscle tissues and liver tissues were sampled. The embryos were carefully taken out from the eggs with tweezers on a certain day. The muscle samples were collected with tweezers and scissors after the skin was removed, then, transferred into frozen pipes immediately and frozen in liquid nitrogen. All samples were kept in −80 °C until RNA extraction. All eggs used in this study were incubated in one incubator with the same condition at the same time. We identified the developmental stages of the embryos by observing the morphology of the head, limb and feather bud. Almost all authors checked the morphology of the embryos to ensure the correct developmental timing. Embryonic sex was determined by PCR amplification of the *Chromo-helicase-DNA-binding 1* (*CHD1*) gene with DNA from the liver as a template [19]. Three breast muscle samples of E13 and E19 female embryos, respectively, were selected for RNA extraction. Total RNA was isolated using TRIzol reagent (Invitrogen, Carlsbad, CA, USA) according to the manufacturer’s instruction. The RNA amount and purity of each sample were quantified (ND-1000, NanoDrop, Wilmington, DE, USA). RNA integrity was assessed (Agilent 2100, Agilent, Beijing, China) with RNA integrity numbers > 7.0. Libraries for miRNA sequencing were prepared using TruSeq Small RNA Sample Prep Kits (Illumina, San Diego, CA, USA) following the manufacturer’s guidelines. Briefly, total RNA was ligated with 3′ and 5′ adaptors. Subsequently, RNA with the adaptors was amplified using reverse transcription PCR followed by gel purification. Finally, the product was subjected to LC Bio (Hangzhou, Zhejiang, China) and sequenced on an Illumina HiSeq 2500 platform according the vendor’s recommended protocol.

### 2.3. Sequencing Data Analysis

All sequencing raw data and processing files were uploaded to the Gene Expression Omnibus using accession number GSE153629 (https://www.ncbi.nlm.nih.gov/geo/query/acc.cgi?acc=GSE153629). Raw reads were subjected to ACGT101-miR (LC Sciences, Houston, TX, USA) to remove adapter dimers, junk, low complexity, common RNA families (rRNA, tRNA, snRNA, snoRNA), and repeats. The prototypic sequences representing repetitive DNA from different eukaryotic species were considered as repeats (http://www.girinst.org/repbase). Sequences with ≥2 N, ≥7 A, ≥8 C, ≥6 G, ≥7 T, ≥10 Dimer, ≥6 Trimer, or ≥5 Tetramer were considered as Junk reads. Then, unique reads of lengths 18–26 nt were blasted against miRBase 22.0, allowing for length variation at both 5′ and 3′ ends and a maximum of one mismatch. Unique hits to mature miRNAs in hairpin arms in miRBase were defined as known miRNAs. The unique sequences mapping to the other arm of the known precursor hairpin, but not the annotated mature miRNA-containing arm, were also defined as known miRNAs. The remaining sequences were mapped to other selected species precursors in miRBase 22.0. To identify genomic locations, all sequences were mapped to the genome of *Anas platyrhynchos* (assembly CAU_duck1.0) using Bowtie 2.4.0 with default parameters [20]. The hairpin RNA structures containing sequences with hairpin RNA structures were predicted using RNAfold software (http://rna.tbi.univie.ac.at/cgi-bin/RNAfold.cgi). The criteria for the secondary structure prediction were as follows: (1) number of nucleotides in one bulge in stem ≤12; (2) number of base pairs in the stem region of the predicted hairpin ≥16; (3) cutoff of free energy (kCal/mol ≤ −15); (4) length of hairpin (up and down stems + terminal loop ≥50); (5) length of hairpin loop ≤20; (6) number of nucleotides in one bulge in mature region ≤8; (7) number of biased errors in one bulge in mature region ≤4; (8) number of biased bulges in mature region ≤2; (9) number of errors in mature region ≤7; (10) number of base pairs in the mature region of the predicted hairpin ≥12; (11) percent of mature bulge in stem ≥80.

A modified global normalization is used to correct the counts of miRNAs among different samples. Basic assumptions and procedures involved in this method are described before [21]. *p*-value was calculated by T-test and Fisher’s exact test. Differential expression of miRNAs based on normalized deep-sequencing counts was analyzed using the criteria |log2 fold change| ≥ 1 and *p* ≤ 0.05.

### 2.4. Naming and Classification of miRNAs

We adopted a unique miRNA nomenclature to clarify the relationship between miRNA in the sequencing data and the reported miRNA. For miRNA that matched with the miRBase, L-n represented a loss of n-bases at the left end of the reported miRNA; R-n represented a loss of n-bases at the right end of the reported miRNA; L + n represented an addition of n-bases at the left end of the reported miRNA; R + n represented an addition of n-bases at the right end of the reported microRNA; 2ss5TC13TA indicated that the fifth base T was replaced by C (ss represents substitution), and at the 13th base T was replaced by A (the initial 2 indicates a total of two bases replaced). New miRNAs begin with PC (predicted candidate) and the position of the 5p or 3p arm was marked.

Based on the blasting result, miRNAs were classified in four groups. Gp1, 2, and 3 were related to the miRNA precursor sequences reported in the miRbase database while Gp4 was not related. Gp1a represented the reads mapped to specific miRNAs/pre-miRNAs in the miRbase, and the pre-miRNAs further mapped to the genome. Gp1b represented the reads mapped to selected (except for specific) miRNAs/pre-miRNAs in the miRbase and the pre-miRNAs further mapped to the genome. Gp2a represented the reads mapped to selected miRNAs/pre-miRNAs in the miRbase, the hit pre-miRNAs did not map to the genome, but the reads were mapped to the genome. Gp2b represents the reads mapped to miRNAs/pre-miRNAs of selected species in the miRbase and the mapped pre-miRNAs were not further mapped to the genome. However, the reads were mapped to the genome; Gp3 represented the reads mapped to selected miRNAs/pre-miRNAs in the miRbase. Neither the mapped pre-miRNAs nor the reads were mapped to the genome; Gp4 represents the reads that did not map to selected pre-miRNAs in the miRbase. However, the reads that mapped to the genome or the extended genome sequences may form hairpins.

### 2.5. Target Gene Prediction and Enrichment Analysis of the Differentially Expressed miRNAs

To predict those genes targeted by the most abundant miRNAs, Target Scan [22] and Miranda [23] were used to identify miRNA binding sites with a context score percentile ≥50 and max energy <−10. Subsequently, data predicted by both algorithms were combined and calculated within certain thresholds. Gene ontology (GO) (http://www.geneontology.org/) and Kyoto Encyclopedia of Genes and Genomes (KEGG) (http://www.genome.jp/kegg/) pathway enrichment analyses were conducted for all the target genes of differentially expressed miRNAs using the DAVID 6.7 functional annotation tool (http://david.abcc.ncifcrf.gov/). All genes annotated in the databases of GO and KEGG were used as background genes. The target genes of differentially expressed miRNAs with *p* ≤ 0.01 were also subjected to protein–protein interaction (PPI) analysis using STRING (https://string-db.org/) v11.0 [24] and the interaction network was adjusted by Cytoscape v3.7.2. All genes of *Gallus gallus* were selected as the background organism since there was no organism of *Anas platyrhynchos* available in STRING.

### 2.6. Oligo Synthesis and qRT-PCR Validation

The specific bulge-loop qRT-PCR primers and mimics for miRNAs were designed and synthesized by RiboBio (RiboBio, Jiangxi, China). The sequence of duck miR-212-5p is 5′-ACCTTGGCTCTAGACTGCTTACT-3′. Total RNA extracted from duck breast muscle was reverse-transcribed using a ReverTra Ace qPCR RT Kit (Toyobo, Osaka, Japan) with specific bulge-loop miRNA qRT-PCR primers. Synthesized samples were then diluted with RNase-free water at a ratio of 1:5. Relative miRNA expression levels were determined by qRT-PCR using the 2 × T5 Fast qPCR Mix (TsingKe, Beijing, China) according to the manufacturer’s instructions. U6 was used as an internal control to normalize the expression levels of miRNAs. The qRT-PCR program was run on an Applied Biosystems (ABI) QuantStudio 5 system (Thermo Fisher, Waltham, MA, USA) using the following protocol: 95 °C for 10 min; 40 cycles of 95 °C for 2 s, 60 °C for 1 min; collection of fluorescence at 65–95 °C. Each sample was performed in triplicate. Relative expression levels were calculated using the 2^−∆∆Ct^ method and presented as mean ± SEM. Student’s T-test was used to compare the significance among different groups.

### 2.7. Cell Culture and Tranfection

The chicken fibroblast cell line DF-1 was cultured in complete Dulbecco’s modified Eagle medium (DMEM; Gibco, Grand Island, NY, USA) containing 10% fetal bovine serum (FBS; Gibco, Grand Island, NY, USA) and 0.2% penicillin/streptomycin (Invitrogen, Carlsbad, CA, USA); it was subsequently incubated at 37 °C in a 5% CO_2_ humidified atmosphere. After growth to confluency, DF-1 was trypsinized at 37 °C for 2 min with 0.25% trypsin (Gibco, Grand Island, NY, USA). Finally, a complete growth medium was added to finish the digestion and cells were collected for passaging or planking. Lipofectamine 3000 reagent (Invitrogen, Carlsbad, CA, USA) was used for cell transfection. DNA plasmids and RNA oligos were transiently transfected into cells.

### 2.8. Dual-Luciferase Reporter Assay

To validate the target relation between miRNAs and functional genes, a dual-luciferase reporter assay was performed using a TransDetect Double-Luciferase Reporter Assay Kit in DF-1 cells following the manufacturer’s protocol. Briefly, after 24 h of 96-well plate seeding, mimic and pmirGLO wild type plasmid, mimic and pmirGLO mutated type plasmid, mimic negative control, and pmirGLO wild type plasmid were co-transfected into DF-1 cells. After 48 h, luminescent signals of firefly and Renilla Luciferase were detected by using a TransDetect Double-Luciferase Reporter Assay Kit on an Infinite 200 PRO plate reader (Tecan Group Ltd., Männedorf, Switzerland). Luciferase activity was presented as mean ± SEM of Firefly luminescence/Renilla luminescence. Three independent experiments were performed, and each experiment included three replicates.

## 3. Results

### 3.1. Overview of Sequencing Data

**Figure 1:**
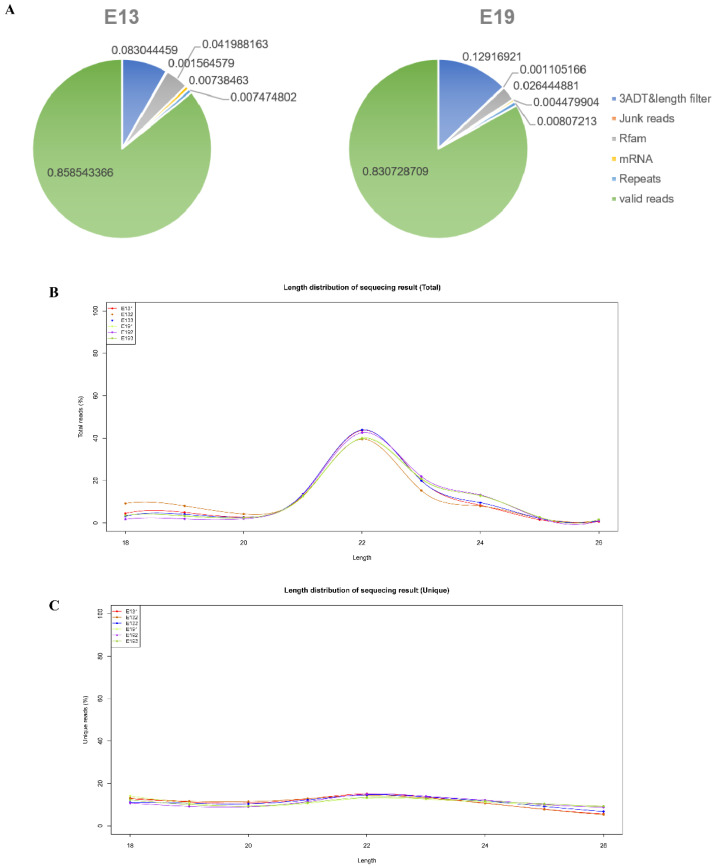
Overview of sequencing data. (**A**) Pie chart distribution annotation of the small RNAs at embryonic day 13 (E13) and E19; (**B**) Length distribution percentage of small RNA sequences in embryonic duck breast muscle. All validated reads of 18–26 nucleotides (nt) for E13 and E19 were assessed for size distribution; (**C**) Length distribution percentage of unique reads in embryonic duck breast muscle. The validated unique reads of 18–26 nt were counted for size distribution.

We selected six embryonic breast muscle samples from two stages to perform miRNA sequencing. A total of 87,906,582 reads were sequenced and 78,321,040 reads were mapped to the *Anas platyrhynchos* genome (Table 1). A total of 74,712,324 validated reads were identified after the elimination of reads with lengths < 18 nt and > 26 nt, and these were matched in the Rfam 14.2 [25,26] (http://rfam.janelia.org) and Repbase [27,28] (http://www.girinst.org/repbase). The ratios of validated reads in E13 and E19 were 85.85% and 83.07%, respectively (Figure 1A). Then, the validated reads were assembled according to samples and 295,703, 355,298, 376,515, 267,865, 236,010, and 329,106 unique reads were found, respectively (Table 1). The length distribution of validated reads of all six samples was counted. The lengths of validated reads were mainly centered on 22 nt and the lengths of most validated reads were concentrated between 21 and 24 nt (Figure 1B). In addition, the lengths of unique reads were also calculated; the number of unique reads at 18 nt to 26 nt was almost identical and the number of unique reads at 22 nt was slightly greater than that at other lengths (Figure 1C).

**Figure 2:**
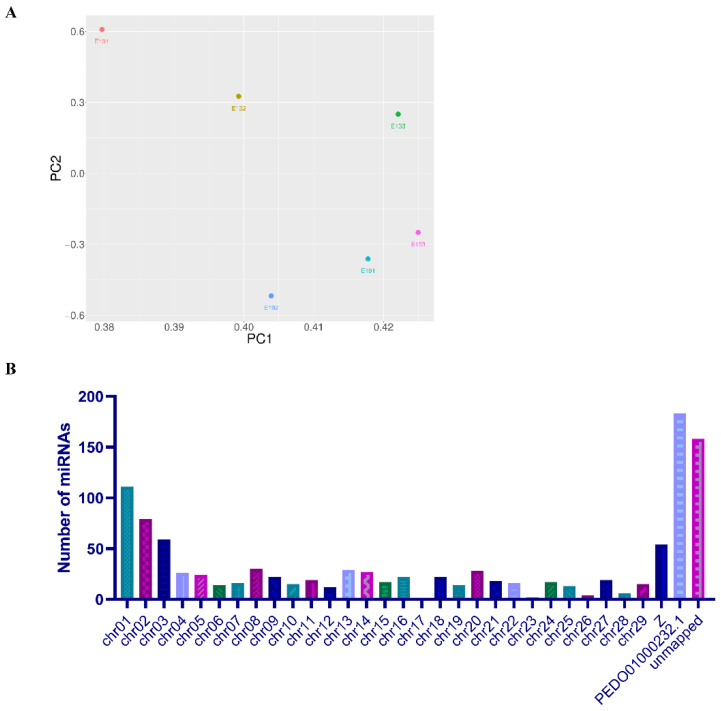
Component analysis of miRNAs in embryonic duck breast muscle. (**A**) PCA plot of miRNAs expressed in each sample. (**B**) Distribution of miRNAs on duck reference chromosomes. PEDO01000232.1: an unplaced scaffold of duck genome sequence.

Based on the normalized miRNA count, principal component analysis (PCA) was conducted using the R package vegan. The results showed that the three samples for the E19 group were close, while the three samples at E13 were more remote, indicating that the miRNA expressions of E13 and E19 were distinct from each other (Figure 2A). Moreover, the miRNAs were mapped to duck chromosomes by blasting with the duck reference genome (http://asia.ensembl.org/Anas_platyrhynchos/Info/Index). Over 85% of miRNAs could be perfectly mapped to the duck genome; however, about 15% could not be found on the duck genomic sequence (Figure 2B). The mapped miRNAs were mainly distributed on chromosomes 1, 2, 3, Z, and an unplaced scaffold of duck genome sequence (PEDO01000232.1) (Figure 2B).

### 3.2. Characterization of Known miRNAs and Novel miRNAs

Based on the mapping results and the blasting results with miRBase 22.0, the conserved known miRNAs were identified from all six embryonic breast muscle samples. The reads did not map to selected pre-miRNAs in the miRbase but did map to the genome or the extended genome sequences; the hairpin second structures were predicted by RNAfold. In this study, a total of 812 known miRNAs were detected as well as 279 novel miRNAs (Supplementary Table S1). The top 25 abundantly expressed miRNAs at E13 and E19 were ordered by the total normalized counts of six samples (Table 2). The most abundant miRNA was hsa-miR-125b, which is a muscle development regulator. Some members of muscle-related miRNA families, including miR-30, miR-206, miR-99, and miR-130, were abundantly expressed in both groups. For most novel miRNAs, the expression level was relatively lower than for known miRNAs. PC-5p-534_9459, with a normalized count of 17,706, had the highest expression among all novel miRNAs (Supplementary Table S2). These results indicated that numerous miRNAs were expressed in embryonic skeletal muscle tissues.

### 3.3. Identification of Differentially Expressed miRNAs

**Figure 3:**
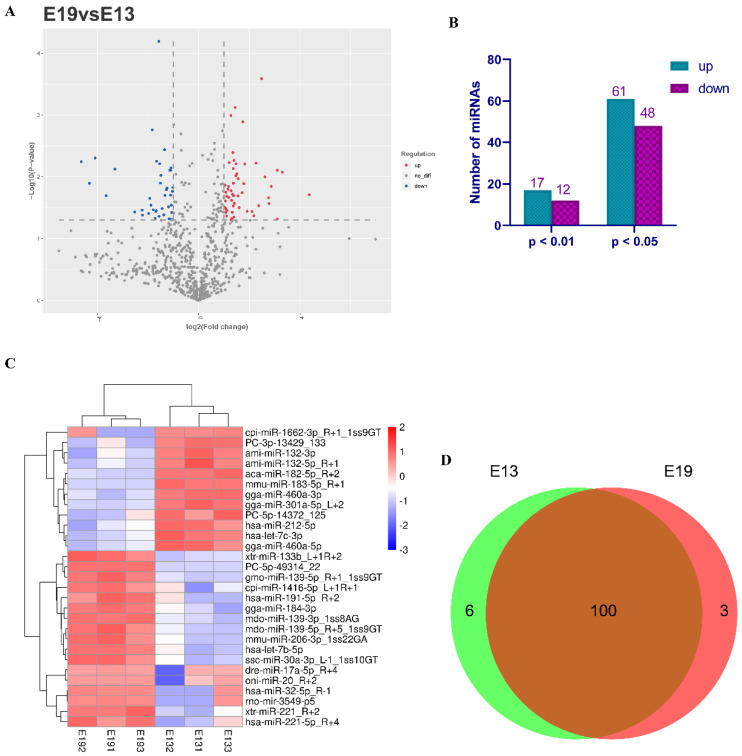
Differentially expressed miRNAs between embryonic day 13 (E13) and E19. (**A**) Volcano chart of miRNAs expressed at E19 vs. E13. Red dots represent up-regulated miRNAs, blue dots represent down-regulated miRNAs, gray dots indicate no difference in expression. (**B**) Numbers of up-regulated and down-regulated miRNAs with *p* < 0.01 and *p* < 0.05. (**C**) Heat map of the differentially expressed miRNAs with *p* < 0.01 at E19 vs. E13 in breast muscle. (**D**) Venn diagrams of differentially expressed miRNAs at E19 vs. E13 in embryonic breast muscle.

To investigate the potential function of the known and novel miRNAs, we performed differential expression analysis with the criteria |log2 fold change| ≥ 1 and *p* ≤ 0.05 (Figure 3A, Supplementary Table S3). A total of 61 up-regulated and 48 down-regulated differentially expressed miRNAs were identified (Figure 3B). With the criteria of |log2 fold change| ≥ 1 and *p* ≤ 0.01, a total of 29 differentially expressed miRNAs were detected, of which 17 were up-regulated and 12 were down-regulated (Figure 3B). Subsequently, we performed clustering analysis on the 29 differentially expressed miRNAs with *p*-values of ≤0.01. The results showed that the up-regulated and down-regulated miRNAs were quite distinct between the E13 and E19 groups and there was little difference among the samples in each group (Figure 3C). Among the 29 differentially expressed miRNAs, PC-5p-49314_22 was an up-regulated novel miRNA, whereas PC-5p-14372_125 and PC-3p-13429_133 were down-regulated novel miRNAs; the remaining 26 miRNAs were known miRNAs. A total of 100 differentially expressed miRNAs were found in both groups; meanwhile, six differentially expressed miRNAs were uniquely expressed in E13 and three were in E19 (Figure 3D). These results demonstrated that miRNAs were differentially expressed during different embryonic muscle stages and may play crucial roles in duck skeletal muscle development.

### 3.4. qPCR Validation of the Differentially Expressed miRNAs

**Figure 4:**
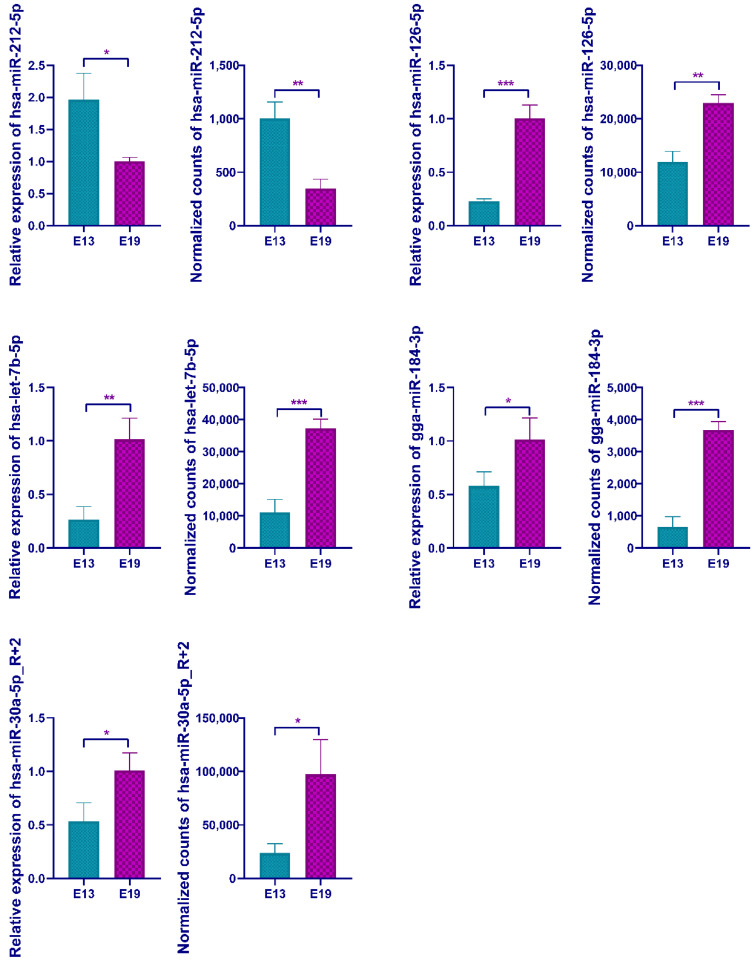
qRT-PCR validation of the identification of differentially expressed miRNAs. In all panels, values are presented as mean ± SEM. * *p* < 0.05; ** *p* < 0.01; *** *p* < 0.01.

To validate the sequencing data, we randomly selected five differentially express miRNAs, hsa-miR-212-5p, has-miR-126-5p, hsa-miR-30a-5p_R + 2, has-let-7b-5p, and gga-miR-184-3p, to perform the qPCR. miRNA expression patterns were in agreement with their sequencing results, in which hsa-miR-212-5p was down-regulated and has-miR-126-5p, hsa-miR-30a-5p_R + 2, has-let-7b-5p, and gga-miR-184-3p were up-regulated (Figure 4). Thus, the deep sequencing data were reliable and efficient for detecting the differentially expressed miRNAs.

### 3.5. Target Gene Prediction and Enrichment Analysis

**Figure 5:**
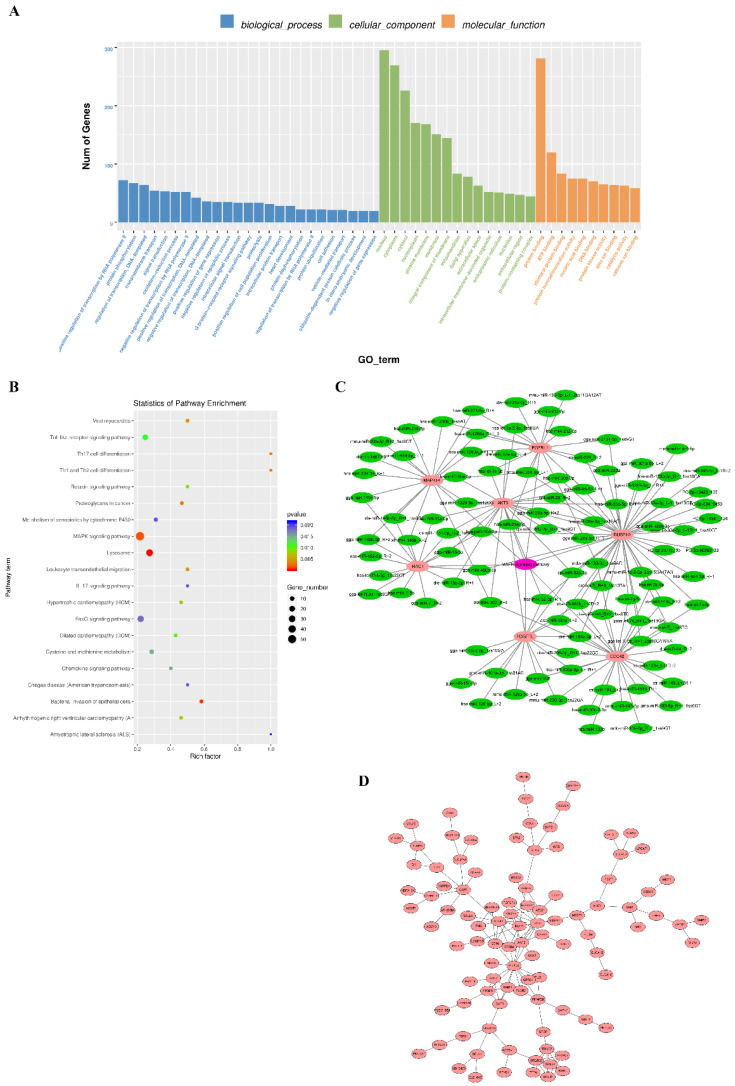
Enrichment analysis of the differentially expressed miRNAs. (**A**) GO function classification of the target genes of differentially expressed miRNAs. The top 25, 15, and 10 GO terms in biological processes, cellular components, and molecular functions, respectively, are shown. (**B**) The top 20 enriched KEGG signaling pathways of the target genes of differentially expressed miRNAs. (**C**) Interaction network of differentially expressed miRNAs and their target genes on MAPK signaling pathways. (**D**) PPI networks for the target genes of differentially expressed miRNAs with a context score percentile >90 and max energy <−25.

Predicting the target genes of miRNAs is important since they exert their biological function by suppressing the translation of their target genes. A total of 20,651 binding sites were identified with all differentially expressed miRNA and 1788 target genes were predicted (Supplementary Table S4). To predict the function of the differentially expressed miRNAs, GO analysis was performed with their target genes. A total of 380 terms in biological processes, cellular components, and molecular functions were significantly enriched for these target genes. The most significantly enriched GO terms included protein binding, ATP binding, nucleus, cytoplasm, cytosol, platelet-derived growth factor receptor signaling pathway, positive regulation of mitogen-activated protein (MAP) kinase activity, and interleukin-1 receptor activity (Figure 5A, Supplementary Table S5). Some muscle-related GO terms, including cardiac muscle tissue morphogenesis, artery smooth muscle contraction, negative regulation of cardiac muscle contraction, and negative regulation of myofibroblast differentiation, were also significantly enriched by target genes of the differentially expressed miRNAs. In addition, KEGG pathway analysis showed that the target genes were notably enriched on lysosomes, bacterial invasion of epithelial cells, and the following pathways: MAPK signaling, FoxO signaling, toll-like receptor signaling, vascular endothelial growth factor (VEGF) signaling, and Hippo signaling (Figure 5B, Supplementary Table S6). Interestingly, MAP kinase related pathways (positive regulation of MAP kinase activity and MAPK signaling pathways) were significantly enriched in different databases. Seven genes that partake in MAPK signaling pathways and the miRNAs targeted to them are listed in Figure 5C. Some myo-miRNAs, including miR-206, miR-15 family, let-7 family, miR-133 family, miR-181 family, and miR-30 family can bind with *AKT Serine/Threonine Kinase 3* (*AKT3*), *FGFRL1*, *Mitogen-activated protein kinase 14* (*MAPK14*), *Platelet derived growth factor receptor like* (*PDGFRL*), *Rac family small gtpase 1* (*RAC1*), *Cell division cycle 42* (*CDC42*), and *Dual specificity phosphatase 10* (*DUSP10*). Furthermore, we also conducted PPI network analysis with 286 target genes with a context score percentile >90 and max energy <−25 (Supplementary Table S7). In the interaction network, some growth regulators, such as *Epidermal growth factor* (*EGF*), *Epidermal growth factor receptor* (*EGFR*), *AKT3*, *Signal transducer and activator of transcription 3* (*STAT3*), and *Phosphatidylinositol-4,5-bisphosphate 3-kinase catalytic subunit alpha* (*PIK3CA*), were found in the center of the cluster (Figure 5D). These results indicated that the differentially expressed miRNAs regulated embryonic skeletal muscle development by targeting the muscle-related genes.

### 3.6. Verification of the Interaction between miRNA and Target Gene

**Figure 6:**
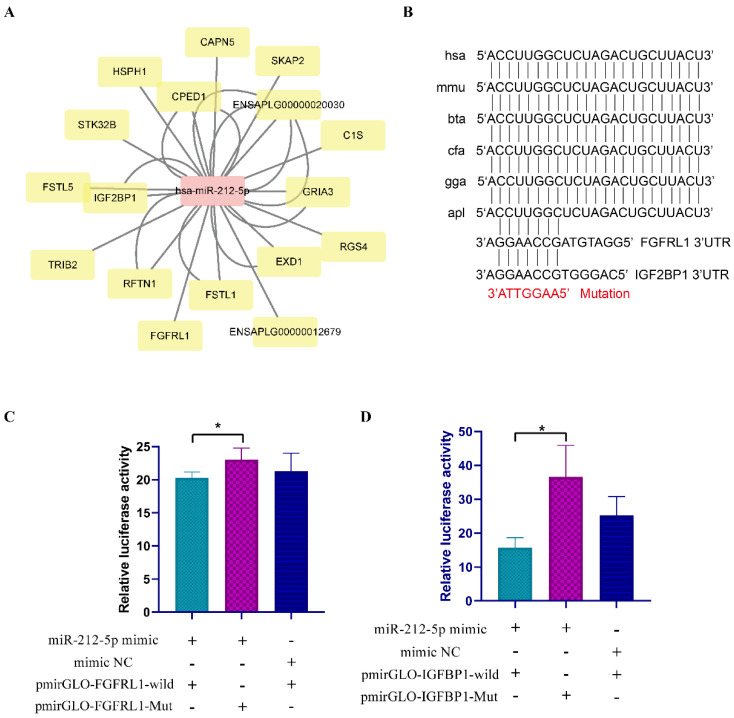
Dual-luciferase reporter assay validates the interaction between hsa-miR-212-5p and target genes. (**A**) has-miR-212-5p and its target genes (TargetScan and Miranda were used for the prediction. Target genes with context score percentile >80 and max energy <−18 were shown). (**B**) Interspecies conservation of miR-212-5p. The potential binding sites on FGFRL1 and IGF2BP1 are shown. The mutated sequence is shown in red. hsa, mmu, bta, cfa, gga, and apl represent *Homo sapiens*, *Mus musculus*, *Bos taurus*, *Canis lupus familiaris*, *Gallus gallus*, and *Anas platyrhynchos*. (**C**,**D**) Relative luminescence was measured and calculated after co-transfecting a wild type or mutant sequence of *FGFRL1* and *IGF2BP1* with miR-212-5p mimic (or mimic NC) in DF-1. In all panels, values are presented as mean ± SEM. * *p* < 0.05.

Based on the bioinformatic results above, we selected hsa-miR-212-5p and its target genes to perform the target relation analysis (Figure 6A). The sequence of miR-212-5p is conserved among species, including humans, mice, cows, dogs, chickens, and ducks (Figure 6B). To validate the binding relation between has-miR-212-5p and *FGFRL1*, *IGF2BP1*, we constructed dual-luciferase reporter vectors by inserting wild-type and mutated-type sequences containing the binding sites of duck *FGFRL1* (NCBI Reference Sequence: XM_005016118.4) and *IGF2BP1* (NCBI Reference Sequence: XM_027445469.1) into pmirGLO plasmids (Figure 6B). After the co-transfection of wild/mutated vector and mimic/mimic NC into DF-1, luminescence signals were tested using an Infinite 200 PRO plate reader. The results showed that the relative luminescence activity of the groups with hsa-miR-212-5p mimic and pmirGLO-FGFRL1-wild was significantly lower than in the groups with hsa-miR-212-5p mimic and pmirGLO-FGFRL1-mut (*p* < 0.05; Figure 6C). Similarly, the relative luminescence activity of the group with hsa-miR-212-5p mimic and pmirGLO-IGF2BP1-wild was less compared with the group which was co-transfected with hsa-miR-212-5p mimic and pmirGLO-IGF2BP1-mut (*p* < 0.05; Figure 6D). These results indicated that *FGFRL1* and *IGF2BP1* target genes of hsa-miR-212-5p.

## 4. Discussion

Ducks are economically viable livestock in China and provide both meat and eggs for human consumption. The Shan Ma duck is a popular small-sized Chinese duck breed that is different from the Pekin duck. A number of studies have shown that miRNAs are related to growth and development in animals [29,30]. In this study, we used high-throughput sequencing to compare the expression of miRNAs in embryonic stages E13 and E19 of the Shan Ma duck, and analyzed the differentially expressed miRNAs, their target genes, and their molecular functions. The results showed that the miRNA lengths were evenly distributed between 18–26 nt, but miRNA expression of 20–24 nt was the highest. The results are similar to other miRNA studies of chickens, ducks, and geese [18,29,31,32]. In the current study, 1091 miRNAs, including 812 known miRNAs and 279 novel miRNAs, were detected; this was a much greater number than in a previous study of Pekin ducks (359 known miRNAs and 23 novel miRNAs) [18]. The expression levels of has-miR-125b-5p, hsa-miR-100-5p_1ss9GT, and hsa-miR-99a-5p_R-1_1ss9GT were the highest in E13 and E19. miR-125b-5p can target *TNF Receptor Associated Factor 6* (*TRAF6*) (a muscular dystrophy factor) to slow down skeletal muscle atrophy [33]. High-throughput miRNA sequencing has shown that miR-100-5p and miR-99a-5p are significantly highly expressed in human skeletal muscle, and could play an important role in skeletal muscle movement by targeting PI3K/AKT signaling pathways [34].

Expression levels of miRNAs are not the same in different tissues or at different developmental stages in same tissue. In this study, we compared miRNAs that were expressed at E13 and E19 and identified 109 differentially expressed miRNAs, of which 61 were up-regulated and 48 were down-regulated. Among these differentially expressed miRNAs, some myo-miRNAs, such as miR-206, members of the let-7 family, miR-16 family, and miR-133 family, already appear in the list of differentially expressed miRNAs. Interestingly, there was a significant difference in the expression of mmu-miR-1a-3p between the two groups, while there was no significant difference and low expression among other members of the miR-1 family, suggesting that the miR-1 family in ducks is less conserved than that of other species. In addition, the expression of gga-miR-460a-3p, gga-miR-184-3p, and xtr-miR221_R + 2 showed extremely significant differences in our data (*p* < 0.0001). miR-184 can regulate cardiac hypertrophy by targeting *Urothelial cancer associated 1* (*UCA1*) [35]. miR-221 can promote the proliferation of satellite cells and inhibit the formation of myotubes [36]. No research on the relationships between miR-460a-3p and muscle development has been reported; therefore, we suggest that it should be closely studied in the future. Regarding novel miRNAs, the expression of PC-5p-534_9459 was very high and there was a significant difference between E13 and E19. Target gene prediction showed that *Retinoic acid receptor beta* (*RARB*), a myoblast differentiation regulator [37], was a target gene of PC-5p-534_9459.

Skeletal muscle development includes myoblast proliferation and the hypertrophy of myotubes. The differentiation of the myoblast and satellite cell is the important process of myogenesis. The myogenesis-related miRNAs, including miR-1a-3p, miR-133a-3p, miR-133b-3p, miR-206-3p, miR-128-3p and miR-351-5p, can negatively regulate the c-Jun N-terminal kinase (JNK)/MAPK pathway and promote myoblast differentiation [38]. Enrichment analysis of the target genes of differential miRNAs showed that these target genes were significantly enriched on 380 GO terms and 38 KEGG signaling pathways. Among the GO terms, 246 biological processes were significantly enriched, including many muscle-related items, cell proliferation items, and cell differentiation items, including positive regulation of MAP kinase activity (*p* < 0.0003). There were also many signaling pathways related to cell proliferation and growth in the enriched signal pathways, among which the MAPK signaling pathway was significantly enriched (*p* < 0.0014). MAPK signaling pathways participate in the signal transduction of various growth factors, cytokines, mitogens, and hormone receptor activation, and play an important role in regulating cell proliferation, growth, and differentiation [39,40,41]. Members of the MAPK signaling pathways, such as *AKT2*, *AKT3*, *RAC1*, and *CDC42*, all play essential roles in muscle development or animal growth [42,43,44,45]. Therefore, we speculate that differentially expressed miRNAs regulate the differentiation of myoblasts and muscle development of duck embryos by targeting MAPK signaling pathway-related genes and regulating the activity of MAPK signaling pathways.

According to the predicted results, we selected target genes with context score percentiles >90 and max energy <−25 to generate an interaction network. In line with expectations, genes related to muscle development appeared in the network. EGF and its receptor, EGFR, play an important role in skeletal muscle development, and synthetic EGF protein can induce the proliferation, differentiation, and myotube fusion of myoblasts [46,47]. Based on the prediction, aca-miR-181a_L + 2, and gga-miR-22-3p can target the *EGF* gene. PIK3CA is an integral part of the PI3K signaling pathway and can interact with AKT and mechanistic target of rapamycin kinase (mTOR) signal pathways; IGF/PI3K/Akt signaling is an important pathway in the regulation of muscle differentiation and growth [48,49]. From our results, we suggest that *PIK3CA* is a target gene of cpi-miR-92b-3p, ssc-miR-30a-3p_L-1R + 1_1ss10GT, and cli-miR-10c-5p_R + 1_1ss11AG. miRNAs can be considered as core to competing endogenous RNAs (ceRNAs) regulatory networks and an intermediate bridge between all types of RNA [10,50]. Based on the prediction results of miRNA and target genes, we constructed a ceRNA network using the same miRNA as the core. We also verified the target relation between miR-212-5p and *FGFRL1*, *IGF2BP1* using luciferase assays. The results indicated that miR-212-5p may regulate skeletal muscle by binding *FGFRL1* and *IGF2BP1*.

## 5. Conclusions

Here, we characterized the miRNA profiles at two different developmental stages of embryonic breast muscle in the Shan Ma duck. We detected 812 known miRNAs and 279 novel miRNAs across six samples. Of all detected miRNAs, a total of 109 miRNAs were differentially expressed between E13 and E19. The GO, KEGG enrichment analysis, and PPI network with the target genes of the differentially expressed miRNAs gave us more clues regarding the underlying mechanisms of the regulation of miRNAs on skeletal muscle development. These results provide a basis for further studies on duck skeletal muscle development.

## Figures and Tables

**Table 1:** Summary of microRNA (miRNA) sequencing data.

Sample	Raw Reads	Mapped Reads	Mapping Percentage	Validated Reads	Unique Reads
E131	14,875,288	13,717,484	92.22%	13,445,305	295,703
E132	13,189,920	11,378,369	86.27%	10,042,092	355,298
E133	18,065,989	16,529,699	91.50%	16,320,782	376,515
E191	11,626,527	9,711,731	83.53%	8,707,352	267,865
E192	13,795,701	12,385,025	89.77%	12,472,937	236,010
E193	16,353,157	14,598,732	89.27%	13,723,856	329,106

**Table 2:** Top 25 most abundant miRNAs in breast muscles of embryonic Shan Ma ducks.

miRNAs	Normalized Counts					
	E131	E132	E133	E191	E192	E193
hsa-miR-125b-5p	891,404	859,062	569,685	381,424	317,414	408,938
hsa-miR-100-5p_1ss9GT	631,277	624,407	471,452	345,984	356,079	419,505
hsa-miR-99a-5p_R-1_1ss9GT	542,627	567,141	448,064	345,143	336,464	387,616
hsa-miR-92a-3p	424,997	675,529	338,265	249,506	212,056	265,128
hsa-miR-140-3p_L-1R + 2_1ss10GT	1,261,316	57,304	318,557	129,118	61,786	109,581
hsa-miR-30c-5p_R + 1	277,921	264,896	237,026	395,513	351,692	357,762
hsa-miR-199a-5p	392,226	260,782	335,561	220,482	246,505	242,580
gga-miR-26a-5p_R + 1	241,562	144,476	224,490	320,746	422,701	315,698
hsa-miR-199a-3p_R-1	284,056	212,832	308,059	212,511	245,447	192,145
hsa-miR-30d-5p_R + 2	181,276	149,487	160,244	232,991	270,104	255,296
hsa-miR-214-3p	366,065	343,733	222,584	108,833	79,526	101,199
gga-miR-206	123,149	175,227	144,083	289,543	273,010	202,096
gga-miR-130b-3p	221,332	229,131	209,359	181,602	176,295	158,693
hsa-miR-363-3p_1ss9GT	147,958	188,728	183,421	179,713	215,735	184,929
hsa-miR-133a-3p_L-1R + 1	72,388	76,236	72,565	257,146	273,573	197,363
mmu-miR-143-3p	99,894	93,780	128,401	178,804	238,750	170,472
hsa-miR-10a-5p_R-1	160,898	141,885	210,035	123,003	92,059	118,950
hsa-let-7g-5p	76,031	94,215	88,589	131,530	165,784	122,125
hsa-miR-148a-3p	96,660	74,660	104,199	101,884	150,264	132,439
hsa-miR-221-3p	66,915	82,560	87,721	79,087	87,797	89,385
mmu-miR-27b-3p_1ss9GT	60,768	67,128	67,252	80,895	120,432	90,534
mmu-miR-181a-5p	89,950	65,948	71,395	82,974	90,517	80,138
gga-miR-191-5p_R-1	61,450	67,512	45,486	105,610	88,121	100,506
gga-miR-2954_R + 1	85,299	123,188	72,930	53,778	38,901	57,196
gga-miR-222a	68,825	81,020	77,822	52,757	50,097	64,669

## Supplementary Materials

The following are available online at https://www.mdpi.com/2076-2615/10/8/1417/s1, Table S1: Summary of known and predicted miRNA in this study. Table S2: List of novel miRNAs in this study. Table S3: List of differentially expressed miRNAs. Table S4: The target prediction result of all differentially expressed miRNAs. Table S5: List of GO enrichment with all target genes. Table S6: List of KEGG enrichment with all target genes. Table S7: List of target genes for PPI network analysis.
